# Lactams Exhibit Potent Antifungal Activity Against Monospecies and Multispecies Interkingdom Biofilms on a Novel Hydrogel Skin Model

**DOI:** 10.1111/apm.13510

**Published:** 2025-01-10

**Authors:** Hafsa Abduljalil, Om Alkhir Alshanta, Safa Chougule, Mark Butcher, Bryn Short, William McLean, Neil Parry, Joanne O'Keeffe, Gordon Ramage

**Affiliations:** ^1^ Oral Sciences Research Group, Glasgow Dental School, School of Medicine, Dentistry and Nursing College of Medical, Veterinary and Life Sciences Glasgow UK; ^2^ Safeguarding Health Through Infection Prevention (SHIP) Research Group, Research Centre for Health, School of Health and Life Sciences Glasgow Caledonian University Glasgow UK; ^3^ Unilever R&D Bebington Wirral UK; ^4^ Penrhos Bio Limited London UK

**Keywords:** *C. albicans*, keratin hydrogel and multispecies biofilms, skin infections

## Abstract

Infections of intact and damaged skin barriers and keratin are frequently associated with complex biofilm communities containing bacteria and fungi, yet there are limited options for successful management. This study intended to focus on the utility of some novel proprietary lactam molecules, quorum sensing (QS)–derived halogenated furanones, which act to block the QS pathway, against key fungal pathogens of the skin ( *Candida albicans* , *Malassezia furfur* and *Microsporum gypseum*). Moreover, we aimed to assess how these actives performed against complex interkingdom biofilms in a clinically relevant model. Two lactam derivatives were tested against a panel of important fungal pathogens and then quantitatively assessed against simple and increasingly complex interkingdom biofilm models on polystyrene coverslips and a novel keratin hydrogel system. The lactams were shown to be effective against a wide range of fungal species in the planktonic and biofilm forms, with no ability to regrow. The fungal component of the multispecies biofilm models was significantly reduced with lactam treatment. Lactam treatment was also comparably effective compared to the non‐prescription topical antifungal ‘Lamisil’ against *C. albicans* early and late biofilms. This study highlights the effectiveness of lactams as a novel antimicrobial for the management of the polymicrobial and interkingdom multispecies biofilms.

## 1 Introduction

In late 2022, the World Health Organization (WHO) published a fungal priority list [1]. The WHO report highlighted the need for better surveillance and treatment options against fungal pathogens that are difficult to detect and that display growing levels of resistance to our key antifungal agents [2]. The estimated morbidity and mortality rates associated with fungi have unsurprisingly made the clinical and academic community stand‐up and take notice [3]. Since then, estimates now suggest that some 6.5 million people are affected by invasive fungal disease, which kill over 2.5 million people annually [4]. Beyond this, fungi impact humans across a broad range of sectors, where their consequences are far reaching, for example, homecare (washing machines), marine (hulls and concrete), built environment, agriculture and the broader healthcare environment [5, 6, 7, 8, 9]. Unequivocally, fungi represent a significant global burden.

Overuse of conventional antifungals in clinical medicine and in agriculture has rendered many antifungals ineffective, such as the azoles and echinocandins [3]. Despite observing a recent renaissance in antifungal development (e.g., Fosmanogepix, Ibrexafungerp, Olorofim, Opelconazole and Rezafungin) [10], there remain significant gaps and opportunities to develop antifungal actives with broad spectrum applicability across different medical and consumer healthcare sectors. For example, worldwide the incidence of skin infections is of significant concern, with dermatophytoses the most common fungal infections that affects almost 20%–25% of the world population [11]. *Malassezia* and *Microsporum* species are common residents of the skin and have the potential to penetrate skin barriers and cause persistently chronic infections. Coupled with the threat from *Candida* species, the predominant human opportunistic yeast has a significant impact in wound environments and then fungi represent a significant challenge [4]. Moreover, the cost of managing wound care is reported to exceed £4.5 billion [12]. Indeed, in the UK it is estimated that the National Health Services (NHS) spend almost 1% of its budget annually on healthcare costs towards ulceration and amputation in diabetic foot ulcers [13], where fungi are an important yet underestimated factor in their clinical management [14]. Therefore, developing new and novel therapeutic approaches to manage fungal infections of the skin and extremities is critically important.

The current arsenal of the available antimicrobial agents for treating skin infection is limited to commonly used agents, such as Bacitracin, Neomycin, Polymyxin, Mupirocin and Fusidic agents, though there is concern for bacterial resistance [15]. Topical antifungal agents include allylamines (e.g., Terbinafine), azoles (e.g., Ketoconazole), Ciclopirox, Tolnaftate, Benzylamine and Amorolfine, and while resistance is rare, there is a tendency in some patient groups for recurrent infections, particularly in those with immunodeficiencies, with particular environmental drivers (humidity), hygiene and antibiotic use [16]. Noteworthy, although the recognised advantages of topical drug delivery in reducing the side effects associated with systemic administration, the required high doses and repeated applications of conventional topical antifungals, especially in immunocompromised patients, can ultimately result in both local and systemic toxicity, in addition to driving antimicrobial resistance [17]. Therefore, there is a continued need to develop new antifungals.

Quorum sensing molecules, small active molecules that have the capacity to signal and interfere with dense microbial biofilm behaviour, are seen as a beacon of light in our fight against infections and antimicrobial resistance (AMR). Lactams (dihydropyrrol‐2‐ones [DHPs]) are considered analogues of fimbrolides (halogenated furanones) that are primarily extracted from the marine red alga *Delisea pulchra* [18]. These compounds have demonstrated to possess an inhibitory effect against *N*‐acyl‐l‐homoserine lactone (AHL)‐mediated quorum sensing systems in many Gram‐negative bacteria, which is essential for bacterial biofilm formation. We and others have previously reported that these quorum sensing molecules have a role in impacting fungal hyphal development [19, 20]. We therefore set out to test the hypothesis whether defined proprietary lactams have broader observable activity beyond bacterial biofilms and instead assess biofilms of fungal origin. Given that bacterial and fungi thrive together in complex interkingdom environments, we hypothesised that we could utilise lactam molecules effectively to manage fungal biomass in simple and complex biofilm communities. In this study, we show for the first time that a lactam molecule can effectively inhibit fungal growth within simple and complex biofilm systems.

## 2 Materials and Methods

### 2.1 Microbial Growth Conditions and Standardisation

Fungal and bacterial strains used in this study are listed in Table S1 and unless stated were obtained from the ATCC culture collection. The *Candida albicans* clinical isolates were obtained from denture stomatitis patients at Glasgow Dental Hospital and School (BC isolates), as previously described [21, 22]. Fungal strains, except *Malassezia furfur*, were maintained on Sabouraud's dextrose agar (SAB; Sigma‐Aldrich, UK) at 30°C for 48 h (*Microsporum gypseum* at 25°C for 5 days). *M. furfur* was maintained on modified Dixon agar at 30°C for 3 days. *Pseudomonas aeruginosa* PA14 and *Staphylococcus aureus* NCTC 10833 were cultured on Luria agar (Sigma‐Aldrich) and incubated aerobically at 37°C for 24 h.

Yeast peptone dextrose broth (YPD; Sigma‐Aldrich) was used to prepare overnight culture for fungal strains (except moulds and *M. gypseum*), and Luria broth (LB; Sigma‐Aldrich) was used for *S. aureus* and *P. aeruginosa*. Fungal cultures were incubated for 18 h at 30°C, while bacterial cultures were incubated aerobically for 18 h at 37°C at 120 rpm in an orbital shaker (IKA KS 4000 i control, Berlin, Germany). Cultured microbial cells were then pelleted by centrifugation and washed twice with PBS. Finally, yeast cells were counted using a Neubauer haemocytometer and bacterial cells standardised using colorimeter to OD_600_ of 0.6, to create equivalent to 1 × 10^8^ cells/mL. Moulds and *M. gypseum* were harvested from agar plates using phosphate buffered saline (PBS; Sigma‐Aldrich) containing 0.025% (v/v) Tween 20 (Sigma‐Aldrich) and counted using a Neubauer haemocytometer.

### 2.2 Planktonic Minimum Inhibitory Concentration Testing (pMIC)

Proprietary lactam compounds (Unilever, Port Sunlight, UK) were prepared in 100% dimethyl sulphoxide (DMSO). Initially, the antimicrobial activity of two lactams, 488 and 491 (chemical analogues) against fungal and bacterial planktonic cells, was determined. The pMIC testing was performed using broth microdilution method according to the M27‐A3 (yeasts) and M38‐A3 (moulds) standard for fungi [23, 24] and the M07‐A10 standard for bacteria [25]. Briefly, fungal cells were adjusted to a cellular density of 2 × 10^4^ cells/mL (2 × 10^3^ cells/mL for moulds) into Roswell Park Memorial Institute (RPMI)‐1640 (Sigma‐Aldrich). RPMI supplemented with oleic acid was used for *M. furfur* [26]. Bacterial cells were adjusted to a density of (2 × 10^5^ cells/mL) into Mueller Hinton Broth (MHB; Sigma‐Aldrich). Serial double‐fold dilutions of each lactam were performed in a 96‐well round‐bottom microtitre plates (Corning Incorporated, Corning, NY, USA) using RPMI, supplemented RPMI or MHB media. Fungal and bacterial cells were added to each active concentration, and the plates were incubated aerobically at 37°C for 24–48 h (or 3 and 5 days for *M. furfur* and *M. gypseum*, respectively). The pMIC concentration was determined as the lowest concentration of each lactam that inhibited visible growth at the bottom of the plate wells. Finally, the fungicidal activity was tested by plating out the growth‐free wells of the pMIC test plates and determining the survivor colony count (CFU/mL). The lactam was considered as fungicidal if it permitted a growth of less than three CFUs. For each pMIC test, at least four technical repeats were used and performed at three different occasions. DMSO controls were used comparative levels to those in lactams and were shown to have no antimicrobial activity.

The kinetics of *C. albicans* SC5314 growth was also assessed in the presence of varying concentrations (0.02–7 μg/mL) of lactam 488. *C. albicans* was standardised to 2 × 10^4^ cells/mL in RPMI medium and added to a 96‐well microtitre plate containing different lactam 488 concentrations. The plate was incubated at 37°C for 18 h in a microtitre plate reader with absorbance measured at 570 nm every 15 min.

### 2.3 Sessile Minimum Inhibitory Concentration (sMIC) Testing of Monospecies Biofilms

For sMIC testing, serial twofold dilutions of lactam 488 were prepared in RPMI and added to preformed 24‐h *C. albicans* SC5341 biofilms and incubated for 24 h. The sMIC_90_ was determined as the lowest lactam concentration that resulted in 90% inhibition of fungal viability as assessed by the (2,3‐bis(2‐methoxy‐4‐nitro‐5‐sulfophenyl)‐2H‐tetrazolium‐5‐carboxanilide) XTT metabolic assay (Fisher Scientific, Paisley, UK), as described previously [27]. To assess the effect of lactam on different stages of biofilm formation, *C. albicans* SC5341 of 1 × 10^6^ cells/mL cellular density was used to develop biofilms over 2, 4, 6, 8 and 24 h. Following each stage of biofilm formation, lactam 488 of concentrations of 7.5, 15, 37.5, 75 and 375 μg/mL was added for additional 24 h. Following incubation with the treatment challenge, the viability of the treated biofilms was assessed by an XTT assay.

Next, biofilm regrowth experiments were performed to assess antifungal tolerance. Early (90 min) and mature (24 h) *C. albicans* SC5314 biofilms were washed with PBS and treated with 480 μg/mL of lactam 488, lactam 491, terbinafine or their combination for 24 h. Following the treatment, the biofilms were washed by gently immersing with PBS and re‐incubated with fresh RPMI medium for an additional 24, 48 and 72 h at 37°C. Untreated controls were also included. After each incubation time, the viability of the biofilms was assessed using XTT assay and the results are expressed as a percentage of viability in relation to untreated controls.

### 2.4 Scanning Electron Microscopy

*C. albicans* SC5314 biofilms were grown on Thermanox coverslips or 90 min or 24 h and treated with 480 μg/mL of lactam 488 for additional 24 h. Following the treatment, the biofilms were washed with PBS, fixed in 2% (v/v) paraformaldehyde, 2% (v/v) glutaraldehyde, 0.15 M sodium cacodylate and 0.15% w/v Alcian Blue and stored at 4°C overnight. Afterwards, the samples were processed, gold sputter coated and imaged using a JEOL JSM‐6400 scanning electron microscope at magnifications ×800 and ×2500.

### 2.5 sMIC Testing Against Multispecies Interkingdom Biofilms on Keratin Hydrogel

To investigate the lactam activity on skin simulated substratum, a hydrogel model system described by our group [28, 29] was modified by adding keratin that was used as a substratum to simulate keratinous skin. The keratin hydrogel (KHG) provides a 3D scaffold that is more representative to the skin than simple Thermanox coverslips. Briefly, 30 μg/mL of keratin powder (Biosynth, UK) was added to the hydrogel components (10% 3‐sulfopropyl acrylate potassium salt, 0.95% poly(ethylene glycol)diacrylate, 0.01% 1‐hydroxycyclohexyl phenyl ketone [Sigma‐Aldrich], 50% heat‐inactivated horse serum [Fisher Scientific] and 2× PBS). The hydrogel mix was then dispensed into a 12‐well plate and irradiated under UV light (366 nm) for 15–20 min or until the gel has visibly set. Keratin hydrogel (KHG) discs were then cut from each well with 2 mm thickness and 13 mm diameter and used as substrate to grow the multispecies biofilms.

For the multispecies biofilm testing, fungi were standardised to 1 × 10^6^ cells/mL ( *C. albicans* SC5314, *M. furfur* and *M. gypseum*) and 1 × 10^7^ cells/mL for bacteria ( *S. aureus* and *P. aeruginosa* ). For all tests on KHG, the microorganisms were standardised in PBS. For tests on Thermanox coverslips, a 1:1 v/v mixture of RPMI:Todd Hewitt broth (THB) (Sigma‐Aldrich) growth media was used for the 3‐ and 4‐species biofilms. This media mixture was used to ensure optimal growth for both fungal and bacterial species. The standardised microbes were then added to the KHG discs or Thermanox coverslips within 24‐well plates to develop mono‐ and multispecies interkingdom biofilms. The 3‐species fungal biofilms were grown for 24 h before being treated with 480 μg/mL of lactam 488 for 24 h. In the case of the 4‐species interkingdom biofilms, dual‐species biofilms of *M. furfur* and *M. gypseum* were first incubated at 30°C for 3 days. Afterwards, *C. albicans* and *S. aureus* were added alongside *M. furfur* and *M. gypseum* dual‐species for 24 h before the treatment. Finally, the treated biofilms were assessed by quantitative PCR.

### 2.6 Quantitative Analysis Using Quantitative Live/Dead PCR

KHG discs and Thermanox coverslips were first washed by PBS, and the attached cells were dislodged by sonication in 1 mL of PBS at 35 kHz for 10 min in an ultrasonic water‐bath (Fisher Scientific). In order to quantify the viable cells within the treated biofilms, a live/dead qPCR technique was used as previously described [30, 31]. Each sample was split into two tubes, 50 μM of propidium monoazide (PMA; Sigma‐Aldrich) was added to one tube, and the samples were incubated in the dark for 10 min. This allows for the PMA uptake and intercalation with the free DNA or the DNA of compromised cells (dead cells). Next, the samples were exposed to 650 W halogen light for 5 min to generate a covalent linkage between the PMA and the DNA and therefore prevent subsequent amplification (detection by PCR). This ensures that all the amplified DNA belongs to the viable cells allowing for the clear quantification of live and dead cells within the samples. DNA was then extracted using MasterPure Yeast DNA Purification Kit (Cambio, UK), as per manufacturer's instructions. The extracted DNA was then amplified using a master mix of Fast SYBR Green (Thermo Fisher Scientific, Paisley, UK), specific forward and reverse primers and RNase‐free water as previously prescribed [32]. The following PCR thermal cycles were applied using Step‐One plus PCR machine (Life Technologies, Paisley, UK); 50°C for 2 min, 95°C for 2 min, 40 cycles of 95°C for 3 s and 60°C for 30 s. Fungal and bacterial DNA was also extracted from 1 × 10^8^ cells/mL cultures to create a standard curve for each microorganism to be used to calculate the colony‐forming equivalents (CFE) in each sample. qPCR primers are listed in Table 1.

**TABLE 1:** Fungal and bacterial primers for qPCR.

Primers	Primer sequences
18S	F‐GAGCGTCGTTTCTCCCTCAAACCGCTGG R‐GGTGGACGTTACCGCCGCAAGCAATGTT
*S. aureus*	F‐ATTTGGTCCCAGTGGTGTGGGTAT R‐GCTGTGACAATTGCCGTTTGTCGT
*P. aeruginosa*	F‐GGGCGAAGAAGGAAATGGTC R‐CAGGTGGCGTAGGTGGAGAA

### 2.7 Comparison of Lactam 488 With Lamisil

*C. albicans* SC5314 early (90 min) and late (24 h) biofilms were grown on KHG as described above. The biofilms were then treated for 24 h with either lactam 488 (480 μg/mL) or Lamisil spray (1% w/w terbinafine hydrochloride, 50 mg/g propylene glycol, 250 mg/g of 96% ethanol, cetomacrogol 1000 and purified water; GlaxoSmithKline, UK). To ensure a valid comparison, lactam 488 was dissolved in the same components present in Lamisil spray (propylene glycol, ethanol, cetomacrogol 1000 and water). Quantitative assessment of killing was then assessed using qPCR. The biofilm biomass of the treated biofilms was assessed using crystal violet (CV) assay as described previously [33]. Briefly, 24 h *C. albicans* SC5314 biofilms were grown and treated with lactam 488 and Lamisil on 24‐well microtitre plates as described above. Next, the biofilms were washed with PBS, left to dry overnight and stained with 0.05% CV (Sigma‐Aldrich). The excess stain was then washed, and 100% ethanol was used to retrieve bound dye. The biomass of the treated biofilms was then quantified spectrophotometrically by reading absorbance at 570 nm using microtitre plate reader.

### 2.8 Statistical Analysis

Graph production and statistical analysis were done using GraphPad Prism (version 7.0 d). Data were tested for normal distribution using D'Agostino–Pearson omnibus normality test. For statistical analysis, two‐tailed Student's *t*‐test was used to compare the means of untreated controls and treated biofilms. One‐way analysis of variance (ANOVA) with Tukey's post‐test was used to compare data of more than two samples.

## 3 Results

### 3.1 Lactams Are Effective Against a Wide Range of Planktonic and Sessile Fungi

Initially, the antimicrobial activity of the lactams 488 and 491 was tested against planktonic fungal cells through planktonic minimum inhibitory concentration (pMIC) tests. The pMIC and minimum fungicidal concentration were established for all strains and are listed in Table 2. Overall, the pMIC range for lactam 488 was observed as 1.87–15 μg/mL and for lactam 491 was 3.75–30 μg/mL. *M. gypseum* was the most sensitive, and the majority of yeasts showing a consistent sensitivity profile at the higher levels stated. *S. aureus* , on the other hand, exhibited sensitivity to both lactam 488 (7.5 μg/mL) and lactam 491 (30 μg/mL), while *P. aeruginosa* demonstrated tolerance to both lactam concentrations up to 240 μg/mL. Furthermore, the fungicidal activity and biofilm inhibition of lactam 488 against the *C. albicans* reference strain were assessed. No visible colonies on the agar plates were detected in wells containing lactam concentrations of 7.5 μg/mL (pMIC) or higher.

**TABLE 2:** Planktonic and sessile minimum inhibitory concentrations.

Fungi	Lactam 488	Lactam 491
*C. albicans* MYA‐2876	7.5	7.5
*C. albicans* ATCC‐10231	7.5	15
*C. albicans* ATCC‐28367	7.5	15
*C. albicans* (*n* = 30)	7.5	15
*C. auris* NCPFs (*n* = 25)	7.5–15	15–30
*Candida glabrata*	7.5	15
*Candida tropicalis*	15	30
*Candida haemolunii*	3.75	7.5
*Candida parapsilosis*	15	30
*Candida krusei*	15	30
*Trichosporon* sp.	15	30
*Rhodotorula* sp.	7.5	15
*Aspergillus fumigatus*	3.75	7.5
*Aspergillus niger*	7.5	7.5
*Scedosporium*	15	15
*Rhizopus*	7.5	15
*Malassezia furfur*	7.5	15
*Microsporum gypseum*	1.87	3.75

**FIGURE 1:**
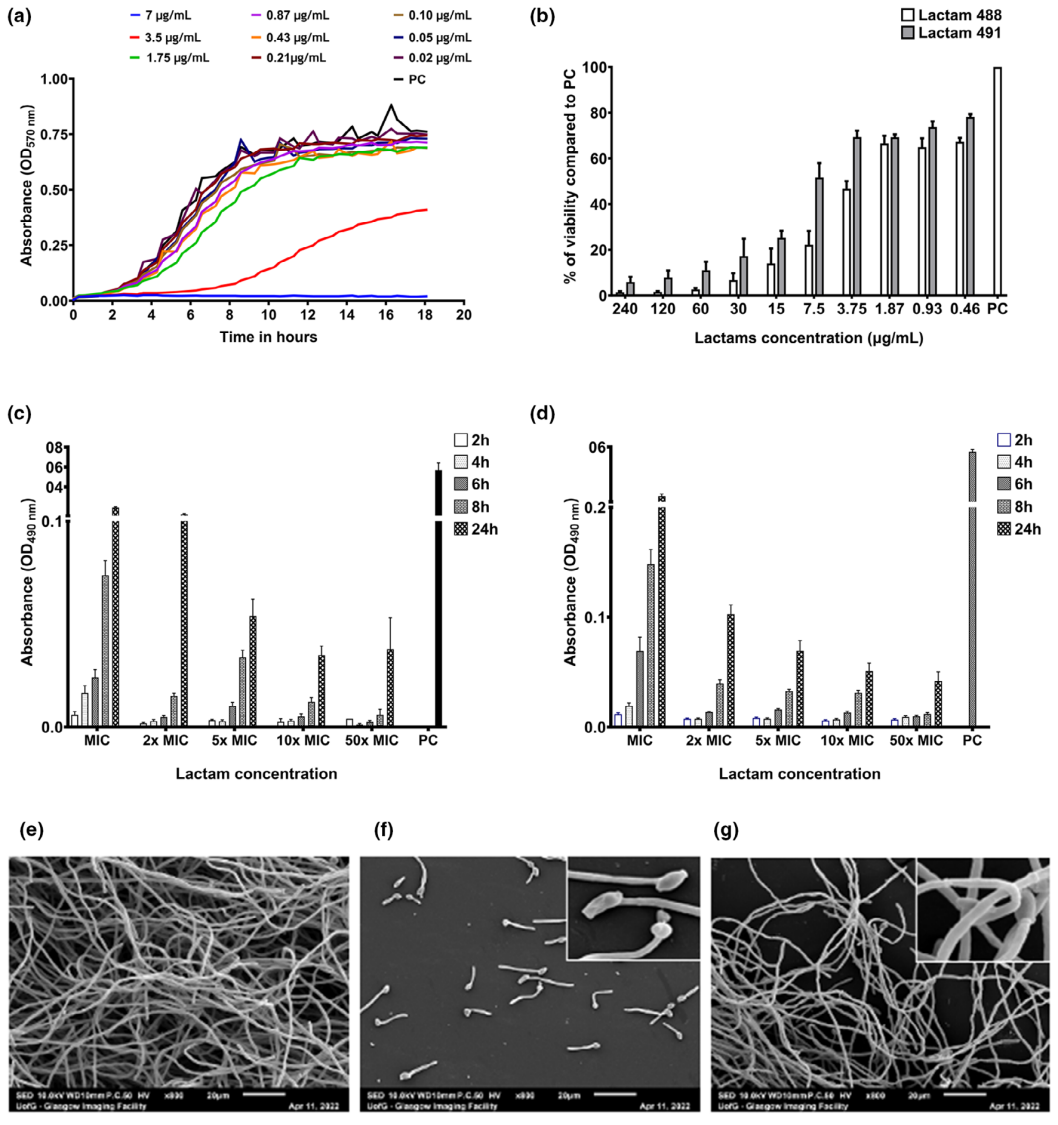
The antifungal activity of Lactams. Growth kinetics of *C. albicans* SC5314 grown in the presence of lactam 488 (a). *C. albicans* planktonic cells were incubated aerobically with different concentrations of lactam 488 at 37°C in a microtitre plate reader with absorbance readings (OD at 570 nm) that were taken every 15 min for 18 h. sMIC of lactams was established using XTT metabolic activity assay against *C. albicans* 24‐h biofilms (b). Assessment of range of lactam concentrations 488 (c) and 491 (d) against different stages of biofilm formation of *C. albicans* by XTT. Each bar represents the mean of data obtained from triplicates of three independent experiments. Error bars represent the standard error of the mean. PC refers to positive untreated control. For SEM imaging, early (90 min) and mature (24 h) *C. albicans* biofilms were also grown on Thermanox coverslips, treated with lactam 488 and imaged. *C. albicans* reference strain 24 h untreated biofilms (e). Early (90 min) *C. albicans* biofilm following 24‐h lactam treatment (f), and mature (24 h) *C. albicans* biofilm following 24‐h lactam treatment (g). Scale bar represents 10 and 20 μm at ×800 and ×2500 magnification, respectively.

Next, biofilm inhibition was assessed using the growth kinetics for *C. albicans* SC5314, where it was clearly shown that concentrations of < 3.5 μg/mL were ineffective against *C. albicans* , whereas at 3.5 μg/mL, there was an intermediate inhibition of growth and a complete retardation of growth at 7 μg/mL (Figure 1a). Next, the sMIC_90_ of both lactams was determined against *C. albicans* reference strain 24‐h biofilms (Figure 1b). The sMIC_90_ was 30 μg/mL for lactam 488 and 120 μg/mL for lactam 491. Afterwards, the effect of lactams on different stages of biofilm formation (2, 4, 6, 8 and 24 h) was assessed using different lactam concentrations (1, 2, 5, 10 and 50× pMIC) (Figure 1c [488], 1d [491]). There was a dose‐dependent effect of both lactams on biofilms, and early stages were more susceptible compared to more tolerant 24‐h biofilm populations. Again, lactam 488 was generally more effective than 491 at the same concentrations. Therefore, lactam 488 was selected for the SEM imaging and the subsequent multispecies biofilm testing.

We further assessed the treated early (90 min) and mature (24 h) biofilms grown on Thermanox coverslip biofilms by SEM imaging. The 24‐h untreated *C. albicans* biofilm showed the typical interlacing networks of hyphae (Figure 1e). Conversely, the lactam 488–treated early biofilm showed dispersed *C. albicans* cells with short hyphae (Figure 1f). This indicates that the fungal cell growth stopped at 90 min shortly upon the initiation of the treatment. The lactam 488–treated mature biofilms showed reduction in hyphal density compared to the untreated control, indicating the ability of this to disturb mature preformed biofilms (Figure 1g).

**FIGURE 2:**
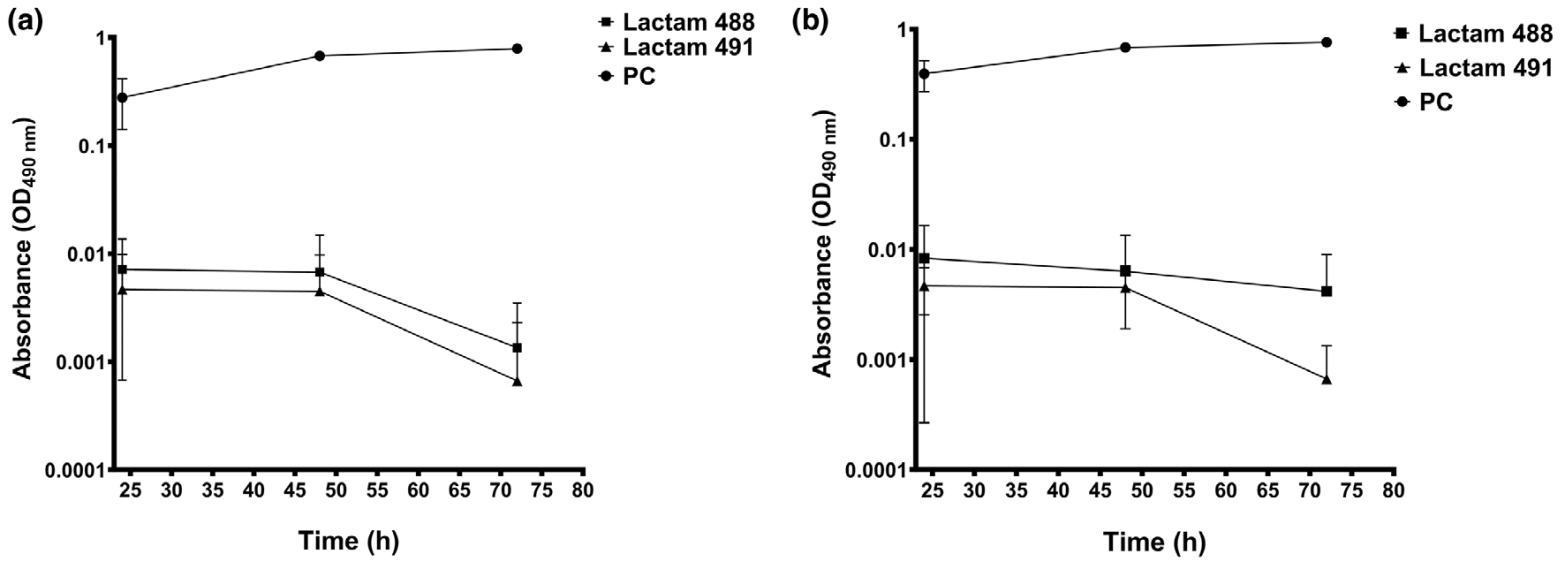
The regrowth potential of *C. albicans* early and mature biofilms treated with lactams. The (a) early (90 min) and (b) mature (24 h) *C. albicans* biofilms were treated with lactam 488 and lactam 491 for 24 h, re‐incubated with fresh medium for 24, 48 and 72 h and biofilm viability assessed by XTT. Values were plotted as log_10_ on the *Y*‐axis. PC refers to positive untreated control.

We next assessed the ability of retarded *C. albicans* cells to regrow following lactam challenge. *C. albicans* SC5314 early (90 min—Figure 2a) and mature (24 h—Figure 2b) biofilms were treated with lactam 488 and lactam 491, for 24 h, and re‐incubated with fresh medium for additional 24, 48 and 72 h. The viability of the treated and re‐incubated biofilms was assessed by XTT, where it was shown that no detectable viability was observed up to 72 h. Together, these data indicate potent long‐lasting antifungal killing.

### 3.2 Lactam 488 Has Broad Antifungal Efficacy Against Complex Biofilms

**FIGURE 3:**
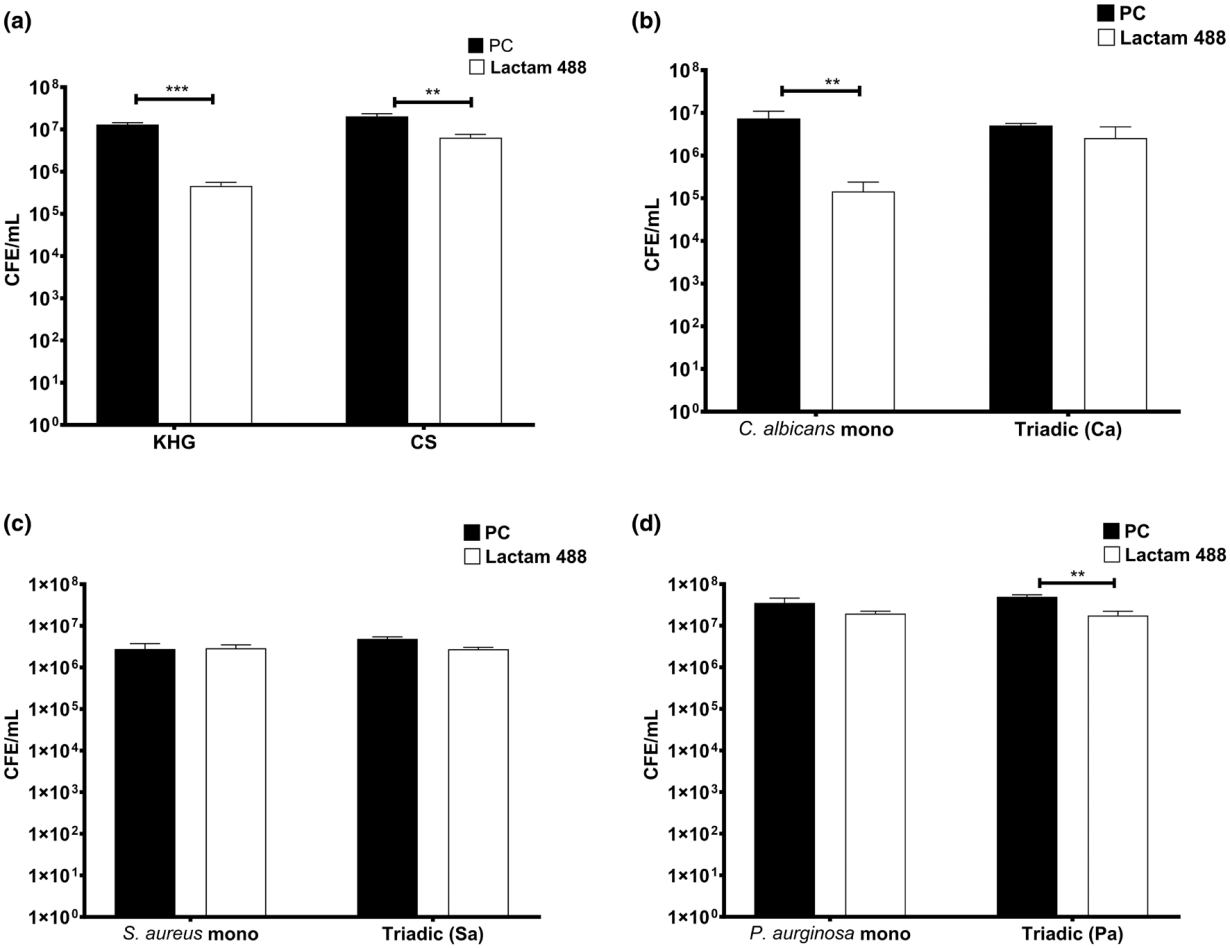
The antifungal activity of lactam 488 against 3‐species biofilm on keratin hydrogel. (a) *C. albicans* 24‐h biofilms were grown on keratin hydrogel or on Thermanox coverslips and treated with lactam 488 for 24 h and assessed by qPCR analysis. Twenty‐four‐hours monospecies and 3‐species biofilms were developed on KHG and treated with lactam 488 for 24 h and assessed by qPCR. (b) *C. albicans* biofilms. (c) *S. aureus* biofilms. (d) *P. aeruginosa* . Each bar represents the mean of data obtained from triplicates of two independent experiments. Error bars represent the standard error of the mean. Statistical significance in CFE/mL between untreated controls and treated biofilms was calculated using a two‐tailed Student's *t*‐test and presented as **p < 0.01, ***p < 0.0001. PC refers to positive untreated control.

To understand how lactam 488 performed in a more complex environment, we created complex biofilms on Thermanox coverslips and on a KHG matrix. Lactam 488 was highly effective against monospecies *C. albicans* 24 h grown onto KHG resulting in 96.4% reduction in CFE/mL compared to untreated control (Figure 3a [*p* > 0.001]). On the Thermanox coverslips (CS), there was also a significant CFE/mL reduction (68.8%, *p* > 0.01). The increased activity of lactam 488 on KHG can be explained by the fact that less fungal cells are attached to KHG compared to the unnatural coverslip substrate. The attached cells on the KHG and coverslips for the untreated controls were 1.3 × 10^7^ CFE/mL and 2 × 10^7^ CFE/mL, respectively.

Next, lactam 488 was tested against our previously optimised 3‐species interkingdom wound model containing *C. albicans* , *S. aureus* and *P. aeruginosa* [28, 34]. Monospecies and 3‐species biofilms of these above were developed onto KHG for 24 h before being treated with lactam 488. Overall, the bacterial members of the interkingdom multispecies, *S. aureus* and *P. aeruginosa* , were more tolerant to the lactam treatment than *C. albicans* . As shown above, lactam 488 was highly effective against *C. albicans* monospecies biofilm, with a significant reduction of 98.1% CFE/mL compared to the untreated control. Within the multispecies biofilms, *C. albicans* became more tolerant to the lactam treatment compared to its monospecies counterpart, though a 50% reduction in the CFE/mL was achievable (Figure 3b). *S. aureus* was less susceptible to the lactam treatment in both mono‐ and triadic‐species biofilms, with a negligible CFE reduction observed despite being sensitive in its planktonic form (pMIC 7.5 μg/mL) (Figure 3c). The effect on *P. aeruginosa* biofilms was more pronounced despite the high planktonic MIC (> 240 μg/mL). Lactam 488 treatment resulted in 45.7% and 65.3% (*p* > 0.01) reduction in *P. aeruginosa* CFE/mL in the monospecies and within multispecies biofilms, respectively (Figure 3d).

**FIGURE 4:**
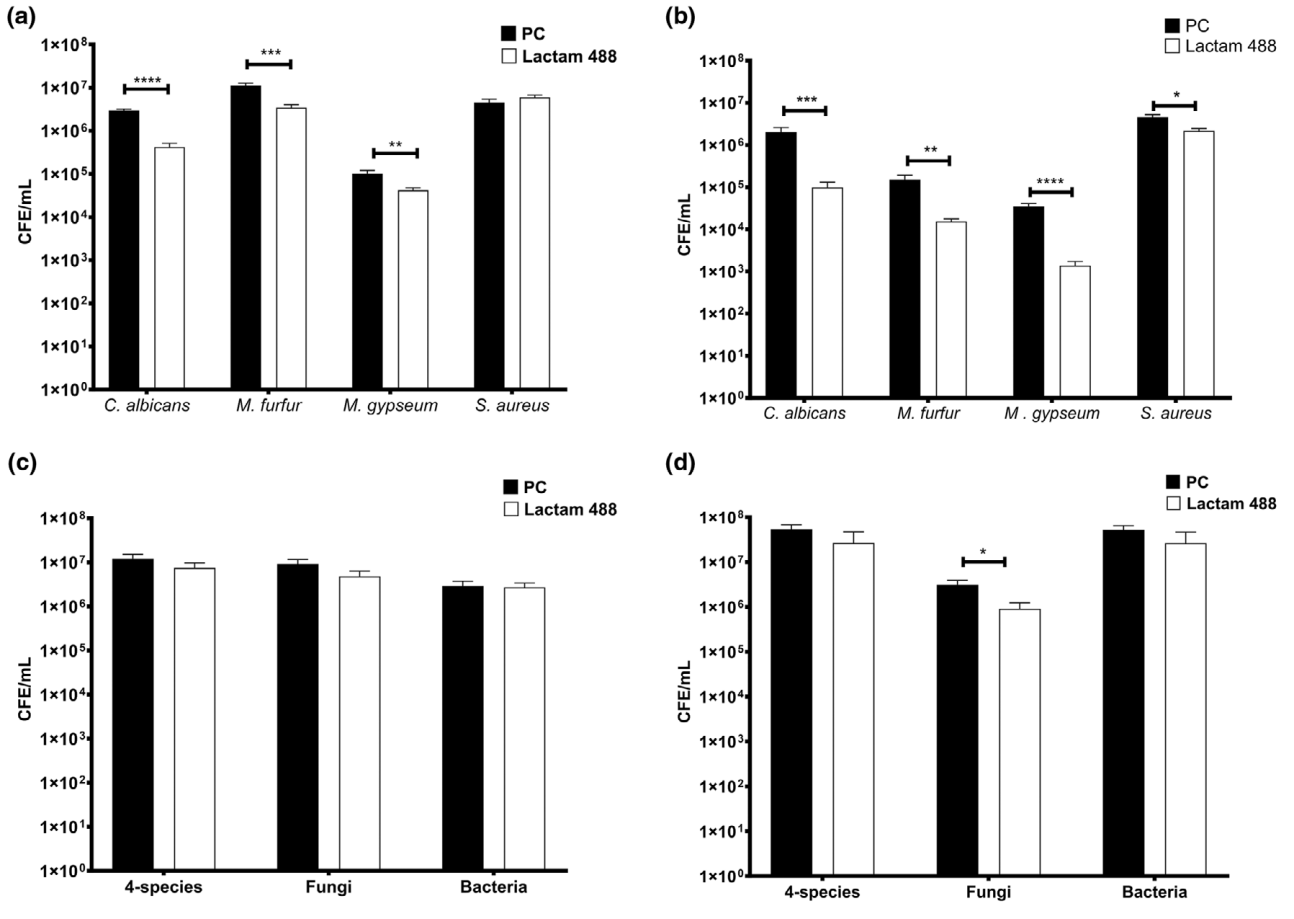
The antifungal activity of lactam 488 against 4‐species biofilm on keratin hydrogel and coverslips. Monospecies and 4‐species biofilms were developed on KHG or coverslips and treated with lactam 488 for 24 h and assessed by qPCR. (a) Lactam‐treated monospecies biofilms of *C. albicans* , *M. furfur*, *M. gypseum* and *S. aureus* on KHG. (b) Lactam‐treated monospecies biofilms of *C. albicans* , *M. furfur*, *M. gypseum* and *S. aureus* on Thermanox coverslips. (c) Lactam‐treated 4‐species biofilms and the corresponding fungal and bacterial communities within the 4‐species biofilms on KHG. (d) Lactam‐treated 4‐species biofilms and the corresponding fungal and bacterial communities within the 4‐species biofilms on Thermanox coverslips. Each bar represents the mean of data obtained from triplicates of three independent experiments. Error bars represent the standard error of the mean. Statistical significance in CFE/mL between untreated controls and treated biofilms was calculated using a two‐tailed Student's *t*‐test and presented as *p < 0.05, **p < 0.01, ***p < 0.001, ****p < 0.0001. PC refers to positive untreated control.

Lactam 488 was also assessed using an 4‐species interkingdom biofilm model. This biofilm model encompasses the most common bacterial skin pathogen, *S. aureus* , and three prevalent fungi associated with the skin, *C. albicans* , *M. furfur* and *M. gypseum*. Mono‐ and multispecies biofilms were developed onto the KHG or on the Thermanox coverslips, which were treated and assessed with qPCR (Figure 4). Overall, lactam 488 resulted in a significant reduction in fungal monospecies cell counts on both substrates, indicating broad antifungal efficacy. On the KHG, *C. albicans* , *M. furfur* and *M. gypseum* showed 85.5%, 69% and 59% CFE/mL reduction, respectively (Figure 4a). Significantly, higher percentages for fungal reduction were observed for fungal biofilms grown on the coverslips with 95.5%, 90% and 95.9% for *C. albicans* , *M. furfur* and *M. gypseum* (*p* > 0.01), respectively (Figure 4b). Interestingly, the KHG harboured significantly higher counts of *M. furfur* than the coverslips. The untreated *M. furfur* contained 1 × 10^7^ CFE/mL compared to only 1.5 × 10^5^ CFE/mL on the coverslips. The same applies to *M. gypseum* counts, albeit less evident, with 1 × 10^5^ CFE/mL and 1.5 × 10^5^ CFE/mL for KHG and coverslips, respectively. As anticipated, *S. aureus* remained relatively tolerant to the treatment on the KHG substratum but showed 53.3% reduction in CFE/mL on the coverslips.

Similar to the pattern observed for the fungal monospecies, lactam 488 was more effective on the overall fungal community within the interkingdom multispecies biofilms grown on the coverslips. There was 48.3% (Figure 4c) reduction and significant 70.6% (*p* < 0.05) (Figure 4d) reduction in fungal CFE/mL for KHG and CS, respectively. Surprisingly, the bacterial count within the multispecies biofilms was significantly higher on the CS (5 × 10^7^ CFE/mL) compared to KHG (2.8 × 10^6^ CFE/mL). The higher bacterial count also elevated the total 4‐species count from 1.2 × 10^7^ CFE/mL for KHG to 5.4 × 10^7^ CFE/mL for the CS. The lactam treatment showed a 38.3% reduction in the 4‐species CFE/mL and minimal bacterial reduction on KHG (Figure 4c). On the CS, the 4‐species reduction was 51.8% and 49% for the bacterial community (Figure 4d).

### 3.3 Lactam 488 Is Equivalent (Noninferior) to the Commercial Antifungal ‘Lamisil’ Against Interkingdom Multispecies Biofilms

**FIGURE 5:**
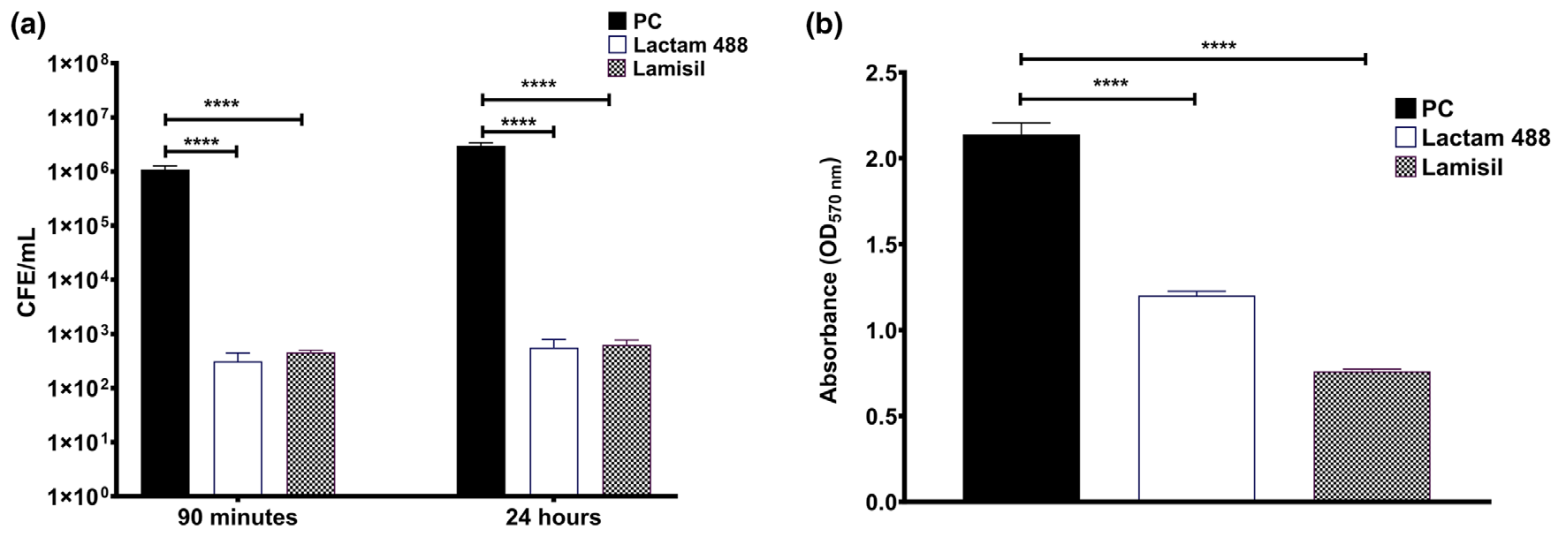
Comparing the antifungal activity of lactam 488 with Lamisil against *C. albicans* biofilm on keratin hydrogel. (a) Monospecies early (90 min) and late (24 h) *C. albicans* biofilms were treated with lactam 488 or Lamisil for 24 h and assessed by qPCR. Each bar represents the mean of data obtained from triplicates of two independent experiments. (b) Twenty‐four hours *C. albicans* biofilms was grown on polystyrene 24 well and treated with the two treatments for 24 h. The biofilm biomass was assessed by the CV assay. Each bar represents the mean of data obtained from triplicates of three independent experiments. Error bars represent the standard error of the mean. Statistical significance between untreated controls and treated biofilms was calculated using one‐way ANOVA with Tukey's post‐test and presented as ****p < 0.0001. PC refers to positive untreated control.

Finally, the lactam 488 antifungal activity against *C. albicans* early and late biofilms was further compared to that of the commercial antifungal product ‘Lamisil’. Lamisil is a very common non‐prescription ‘over the counter’ antifungal product for the management of tinea pedis (Athlete's foot) containing 1% terbinafine as the active ingredient. *C. albicans* early (90 min) and mature (24 h) biofilms were grown on KHG and treated with lactam 488 or Lamisil for 24 h and results assessed by qPCR (Figure 5). Both treatment arms were significantly effective against the *C. albicans* monospecies biofilms at both time points with CFE/mL reduction of > 99% (*p* > 0.0001) (Figure 5a). There was no significant difference between the two treatments at both time points. We also assessed and compared the ability of the two treatments to disturb the preformed 24 h *C. albicans* biofilms. Both treatments significantly reduced the biofilm biomass compared to untreated controls as measured by the CV assay (*p* > 0.0001) (Figure 5b).

## 4 Discussion

Skin and nail infections are a very common public health issue with an increased incidence worldwide and are also associated with high morbidity and mortality [35, 36]. The high recurrence rate of these infections and the limitations associated with the current treatment modalities such as microbial resistance, toxicity and drug interactions highlight the need for new antimicrobial agents [37, 38]. This study was focused on chronic wounds (e.g., diabetic foot ulcer) and fungal infections of the skin, where it was shown that lactam compounds have the capacity to inhibit and kill fungi in simple and complex biofilm model systems.

We assessed the antimicrobial activity of potential novel antimicrobial agents, lactams, against monospecies, 3‐species ( *C. albicans* , *S. aureus* and *P. aeruginosa* ) and 4‐species ( *S. aureus* , *C. albicans* , *M. furfur* and *M. gypseum*) models, as well as a panel of well‐known fungal pathogens. Here we report for the first time the potent antifungal activity of the lactams against several fungal species. To further elucidate the effectiveness of lactams, the antibiofilm activity was assessed against *C. albicans* at different time points of biofilm formation. It was revealed that lactam 488 is more potent than lactam 491 within the same concentration range. Therefore, lactam 488 was utilised for further antimicrobial testing for multispecies models. Notably, no regrowth was detected, even with prolonged re‐incubation in fresh media after lactam treatment. This is in contrast with what was demonstrated previously with well‐known powerful disinfectants agents as sodium hypochlorite [22, 32] and chlorhexidine [39]. This demonstrates that tolerant microbial populations have the ability to regrow following the initial treatment with these disinfectants, which may contribute to infections recurrence [40, 41]. Here, lactams have a superior mode of action by irreversibly inhibiting their propagation on surfaces. This substantiative effect is subject to further study.

To mimic the keratinous skin infections, microbes were grown on 3D keratin hydrogel, a 3D model which was developed previously by our group to mimic the skin substratum with keratin added. To ensure that lactams are equally functional on the KHG, the effect of lactam was tested against biofilms of *C. albicans* grown on both KHG and coverslips. Lactam 488 showed more killing ability against biofilms grown on KHG compared to the coverslips. This partly can be explained by the fact that less fungal cells were attached to KHG compared to the coverslips representing less challenging biofilm for lactam to affect. These coverslips are surface‐treated to promote cell adhesion and a growth media was used to support the growth of microbes, whereas in the case of KHG, the only nutrient source was the KHG incorporated serum. Moving forward, our optimised triadic model of multispecies biofilm containing *P. aeruginosa* and *S. aureus* , the two most co‐colonisers of chronic wounds, and *C. albicans* was tested. Interestingly, the bacterial components of the multispecies model were more tolerant to lactam treatment in comparison with fungi. This indicates that lactams are more effective on fungi and may confirm the need for more representable polymicrobial biofilm models to effectively assess the response of chronic infections to the tested antimicrobial agents. Indeed, we have data to indicate a wide range of antimicrobial sensitivity amongst bacterial species to lactams, which suggests our particular strains are protected by fungal extracellular polymers (diffusion issue). Indeed, this has been shown in studies of vancomycin with dual species *C. albicans* and *S. aureus* populations, which cover themselves in matrix to prevent access of vancomycin [42, 43].

Considering the potent antifungal activity of lactam, the 4‐species model was developed incorporating key fungal skin pathogens, *C. albicans* , *M. furfur* and *M. gypseum*, along with the most common skin coloniser bacteria, *S. aureus* . There was a significantly higher growth of *M. furfur* and to a lesser extent *M. gypseum* on the KHG compared to the coverslips. The high affinity of the dermatophyte, *M. gypseum* to the keratin and the lipophilic nature of *M. furfur*, may account for the increased number of these fungal cells adhering to the KHG. It was demonstrated that lactam had a significant antifungal activity on both KHG and coverslip substrates. However, in the multispecies model grown on CS, there was greater reduction in cell counts in both the bacterial and fungal component when compared to the KHG counterpart. This is in agreement with our previous study simulating the chronic wound, where a greater tolerance to wound washes was observed when triadic biofilms were grown on KHG compared to biofilms grown on plastic 2D surfaces [28]. The use of skin‐simulated models as opposed to the plastic surfaces has clearly shown that both microorganisms' growth and their response to the treatment are significantly influenced by the substrate and the nutrient source used in the experimental setting.

Lactams as potential antifungal compounds with comparable in vitro efficacy to ‘Lamisil’ demand further investigations. Studying the in vivo activity, mechanism of action, combined active interaction and the potential of the treated pathogens to develop resistance to lactams appear to be the logical steps to follow. Lactams have the potential to be used in broad health‐related and environmental applications ranging from topical applications to treat skin infections to surface coatings and disinfection. We can greatly benefit from the advances in the sequencing technologies and employ next‐generation sequencing to further elucidate the response of microorganisms to the lactams and their mechanism of action. Indeed, we are currently exploring its activity in highly complex microbiome populations to assess its broad antimicrobial activity. There are limitations to our approach through using single bacterial species in the model, but this proof‐of‐concept study provides solid data to support its use against fungal communities, but also necessitates further work in a wide array of bacterial biofilm applications.

In conclusion, this study investigates the antimicrobial effect of novel quorum sensing blockers, lactams, with special focus on skin infections using multispecies models. In the light of the limited number of the available antifungal agents, the increased rates of drug resistance and the high recalcitrance of skin infections, the need for new antimicrobials and new drug targets arise. The results present herein offer a proof of concept and starting point to trigger further investigations on lactams as potential novel antimicrobial agents with wide range of applications.

## Conflicts of Interest

N.P. and J.O. are employees of Unilever, and N.P. has financial interests in PenrhosBio.

## Supporting information


**Figure S1.** Gene copies of specific bacteria and 16S rRNA using droplet digital PCR (ddPCR).
**Figure S2**. Number of reads classified for each bacterium detected in samples spiked with an ATCC‐MSA‐2002 mock community.
**Figure S3**. The proportion and number of reads classified for each bacterium detected in the ATCC‐MSA‐2002 mock community.
**Table S1**. Mean value of droplet digital PCR (ddPCR) gene copies/μL of *nuc*, *uidA* and 16S rRNA. genes, in a DNA‐extracted ATCC‐MSA‐2002 mock community and run in triplicates.
**Table S2**. Sequence data analysis of samples spiked with *S. aureus* , *E. coli* or *S. pneumoniae* .
**Table S3**. sequencing data analysis of samples spiked with ATCC‐MSA‐2002 mock community.

## Data Availability

The data that support the findings of this study are available on request from the corresponding author. The data are not publicly available due to privacy or ethical restrictions.
