# Supplementary material for: Lactams Exhibit Potent Antifungal Activity Against Monospecies and Multispecies Interkingdom Biofilms on a Novel Hydrogel Skin Model
Source: APMIS. 2025 Jan 10;133(1):e13510. doi: 10.1111/apm.13510 (PMC11718591; doi:10.1111/apm.13510)
Supplement: Supplementary file 1 — Figure S1. Gene copies of specific bacteria and 16S rRNA using droplet digital PCR (ddPCR). Figure S2. Number of reads classified for each bacterium detected in samples spiked with an ATCC‐MSA‐2002 mock community. Figure S3. The proportion and number of reads classified for each bacterium detected in the ATCC‐MSA‐2002 mock community. Table S1. Mean value of droplet digital PCR (ddPCR) gene copies/μL of nuc, uidA and 16S rRNA. genes, in a DNA‐extracted ATCC‐MSA‐2002 mock community and run in triplicates. Table S2. Sequence data analysis of samples spiked with S. aureus , E. coli or S. pneumoniae . Table S3. sequencing data analysis of samples spiked with ATCC‐MSA‐2002 mock community. [file APM-133-0-s001.docx]

Supplementary table 1: Minimum inhibitory concentrations (µg/mL) of planktonic fungi and bacteria to Lactams 488 and 491.

| **Microorganism** | **Lactam 488** | **Lactam 491** |
| --- | --- | --- |
| ***C. albicans*** |  |  |
| *C. albicans* SC5314 | 7.5 | 7.5 |
| *C. albicans* ATCC 10231 | 7.5 | 15 |
| *C. albicans* ATCC 1043 | 7.5 | 15 |
| *C. albicans* ATCC 1384 | 7.5 | 15 |
| *C. albicans* ATCC 3153A | 7.5 | 15 |
| *C. albicans* BC009 | 7.5 | 15 |
| *C. albicans* BC013 | 7.5 | 15 |
| *C. albicans* BC020 | 7.5 | 15 |
| *C. albicans* BC023 | 7.5 | 15 |
| *C. albicans* BC037 | 7.5 | 15 |
| *C. albicans* BC038 | 7.5 | 15 |
| *C. albicans* BC039 | 7.5 | 15 |
| *C. albicans* BC043 | 7.5 | 7.5 |
| *C. albicans* BC044 | 7.5 | 7.5 |
| *C. albicans* BC045 | 3.75 | 7.5 |
| *C. albicans* BC097 | 7.5 | 15 |
| *C. albicans* BC098 | 7.5 | 15 |
| *C. albicans* BC099 | 7.5 | 15 |
| *C. albicans* BC106 | 7.5 | 15 |
| *C. albicans* BC107 | 7.5 | 15 |
| *C. albicans* BC108 | 7.5 | 15 |
| *C. albicans* BC115 | 7.5 | 15 |
| *C. albicans* BC116 | 7.5 | 15 |
| *C. albicans* BC117 | 7.5 | 15 |
| *C. albicans* BC127 | 7.5 | 15 |
| *C. albicans* BC128 | 7.5 | 15 |
| *C. albicans* BC129 | 7.5 | 15 |
| *C. albicans* BC135 | 7.5 | 15 |
| *C. albicans* BC136 | 7.5 | 15 |
| *C. albicans* BC137 | 7.5 | 15 |
| *C. albicans* BC145 | 7.5 | 15 |
| *C. albicans* BC146 | 7.5 | 15 |
| *C. albicans* BC159 | 7.5 | 15 |
| *C. albicans* BC160 | 7.5 | 15 |
| ***C. auris*** |  |  |
| *C. auris* NCPF 182 | 15 | 15 |
| *C. auris* NCPF 8977 | 15 | 30 |
| *C. auris* NCPF 8991 | 15 | 15 |
| *C. auris* NCPF 8996 | 15 | 15 |
| *C. auris* NCPF 174 | 15 | 30 |
| *C. auris* NCPF 166 | 7.5 | 15 |
| *C. auris* NCPF 8990 | 15 | 30 |
| *C. auris* NCPF 13004 | 7.5 | 15 |
| *C. auris* NCPF 8978 | 15 | 30 |
| *C. auris* NCPF 8980 | 15 | 30 |
| *C. auris* NCPF 8983 | 15 | 15 |
| *C. auris* NCPF 8986 | 15 | 15 |
| *C. auris* NCPF 8993 | 7.5 | 15 |
| *C. auris* NCPF 13026 | 15 | 30 |
| *C. auris* NCPF 13016 | 15 | 30 |
| *C. auris* NCPF 139 | 15 | 30 |
| *C. auris* NCPF 165 | 15 | 30 |
| *C. auris* NCPF 199 | 15 | 30 |
| *C. auris* NCPF 206 | 7.5 | 15 |
| *C. auris* NCPF 13005 | 15 | 15 |
| *C. auris* NCPF 13015 | 15 | 15 |
| *C. auris* NCPF 8973 | 7.5 | 15 |
| *C. auris* NCPF 185 | 7.5 | 15 |
| *C. auris* NCPF 8984 | 7.5 | 15 |
| *C. auris* NCPF 8989 | 7.5 | 15 |
| **Non-albicans *Candida*** |  |  |
| *Candida glabrata* WT2001 | 7.5 | 15 |
| *Candida tropicalis* BC064 | 15 | 30 |
| *Candida haemolunii** | 3.75 | 7.5 |
| *Candida parapsilosis** | 15 | 30 |
| *Candida krusei* NCPF 3953 | 15 | 30 |
| **Other yeasts** |  |  |
| *Trichosporon spp.** | 15 | 30 |
| *Rhodotorula spp.** | 7.5 | 15 |
| *Malassezia furfur** | 7.5 | 15 |
| **Moulds** |  |  |
| *Aspergillus fumigatus Af293* | 3.75 | 7.5 |
| *Aspergillus niger** | 7.5 | 7.5 |
| *Scedosporium spp.** | 15 | 15 |
| *Rhizopus spp.** | 7.5 | 15 |
| **Others** |  |  |
| *Microsporum gypseum** | 1.87 | 3.75 |

*Clinical isolate from Ramage culture collection
